# Mechanisms of diabetic foot ulceration: A review

**DOI:** 10.1111/1753-0407.13372

**Published:** 2023-03-09

**Authors:** Haibo Deng, Binghui Li, Qian Shen, Chenchen Zhang, Liwen Kuang, Ran Chen, SiYuan Wang, ZhiQiang Ma, Gongchi Li

**Affiliations:** ^1^ Department of Wound Repair, Liyuan Hospital Affiliated to Tongji Medical College Huazhong University of Science and Technology Wuhan Hubei China; ^2^ School of Foreign Studies Zhongnan University of Economics and Law Wuhan Hubei China; ^3^ Department of Hand Surgery, Union Hospital affiliated to Tongji Medical College Huazhong University of Science and Technology Wuhan Hubei China

**Keywords:** diabetic foot ulcer, wound healing, epigenetics, physiology and pathology, signaling pathway, 糖尿病足溃疡, 创面愈合, 表观遗传学, 生理病理, 信号通路

## Abstract

Diabetic foot ulcers (DFUs) are associated with complex pathogenic factors and are considered a serious complication of diabetes. The potential mechanisms underlying DFUs have been increasingly investigated. Previous studies have focused on the three aspects of diabetic peripheral vascular disease, neuropathy, and wound infections. With advances in technology, researchers have been gradually conducting studies using immune cells, endothelial cells, keratinocytes, and fibroblasts, as they are involved in wound healing. It has been reported that the upregulation or downregulation of molecular signaling pathways is essential for the healing of DFUs. With a recent increase in the awareness of epigenetics, its regulatory role in wound healing has become a much sought‐after trend in the treatment of DFUs. This review focuses on four aspects involved in the pathogenesis of DFUs: physiological and pathological mechanisms, cellular mechanisms, molecular signaling pathway mechanisms, and epigenetics. Given the challenge in the treatment of DFUs, we are hopeful that our review will provide new ideas for peers.

## Graphical abstract

**Highlights**

- With the increase in the elderly population, diabetic foot ulcers (DFUs) severely affect human health and societies worldwide. They will pose significant medical, social, and socioeconomic challenges.
- Reviewing the existing studies on the pathogenesis of DFUs, most of them focus on clinical nursing and treatment. However, the molecular mechanisms of DFUs are poorly studied.
- The mechanisms of inflammatory response, signal pathway, and epigenetics in DFUs are highlighted.
- The authors suggest reasonable choices of finding out important targets for treatment of disorders of DFUs and developing the effective products underlying the molecular pathogenesis of DFUs.
- Future insights into the use of motor molecules to carry drug molecules in combination with existing materials are briefly discussed.

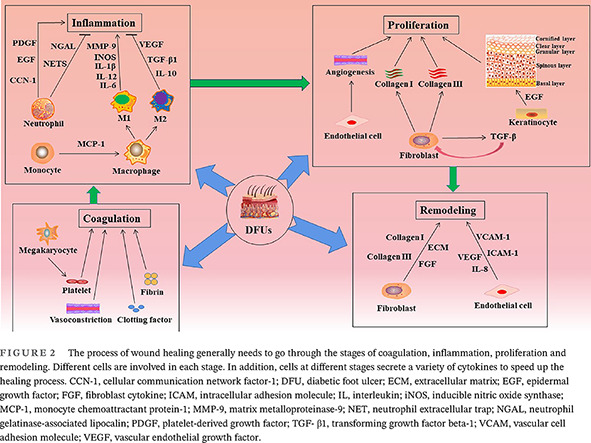

## 摘要

糖尿病足溃疡(DFUs)与复杂的病理因素有关，被认为是糖尿病的严重并发症。目前，潜在的DFUs机制已经越来越受到研究关注。以往的研究主要集中在糖尿病周围血管疾病、神经病变和创伤感染三个方面。随着技术的进步，研究人员逐渐开始使用免疫细胞、内皮细胞、角质细胞和成纤维细胞进行研究，因为它们参与了创伤愈合过程。报道称，分子信号通路的上调或下调对DFUs的愈合至关重要。随着对表观遗传学认识的增强，它在创伤愈合中的调控作用成为治疗DFUs的热门趋势。本综述关注DFUs发病机制涉及的四个方面:生理和病理机制、细胞机制、分子信号通路机制和表观遗传学。鉴于DFUs治疗的挑战，我们希望本综述能为同行提供新思路。

## 1 BACKGROUND

With the aging of the global population, there has been an increase in the number of patients with diabetes every year. The statistics in 2019 projected 135.6 million individuals among the 65–99‐year‐old population worldwide to be diabetic, and this number is expected to reach 195.2 million by 2030, making it the largest global epidemic in the 21st century.^1^ The global prevalence of diabetic foot ulcers (DFUs) is about 6.4% in the diabetic population.^2^ About 50%–60% of patients with DFUs will develop diabetic foot infection (DFI) and 15% will undergo amputation. The 5‐year risk of death in patients with DFUs is 2.5 times that in patients without foot ulcers.^3^ The global direct health expenditure in diabetes was about $700 billion in 2019 and is estimated to increase to $825 billion by 2030, of which the medical costs related to DFUs will account for a third of the total costs incurred in the management of diabetes.^4^ Currently, patients with DFU suffer physical, mental, and economic trauma caused by this debilitating condition. Keeping in mind the urgent needs of patients and society, several researchers have begun to focus on research related to DFUs, as the study of its pathogenesis is key in treatment.

The normal wound‐healing process mainly involves the following four stages: (a) in the “hemostasis” stage, vasoconstriction, platelet aggregation, and recruitment of circulating coagulation factors in the wound occur; (b) in the “inflammation” stage, inflammatory cells gather and secrete inflammatory factors; matrix metalloproteinase (MMP)‐9 is secreted by macrophages and neutrophil extracellular reticular traps (NETs) are secreted by neutrophils; (c) in the “proliferation” stage, the inflammation subsides and skin cells, such as keratinocytes secreting epidermal growth factor (EGF), proliferate and migrate to the wound bed; and (d) in the “remodeling” stage, new tissue is remolded and deposited through the extracellular matrix and neovascularization, involving fibroblasts that secrete fibroblast cytokines (FGF) and vascular endothelial cells that secrete vascular endothelial growth factor (VEGF).^5^, ^6^, ^7^

**FIGURE 1:**
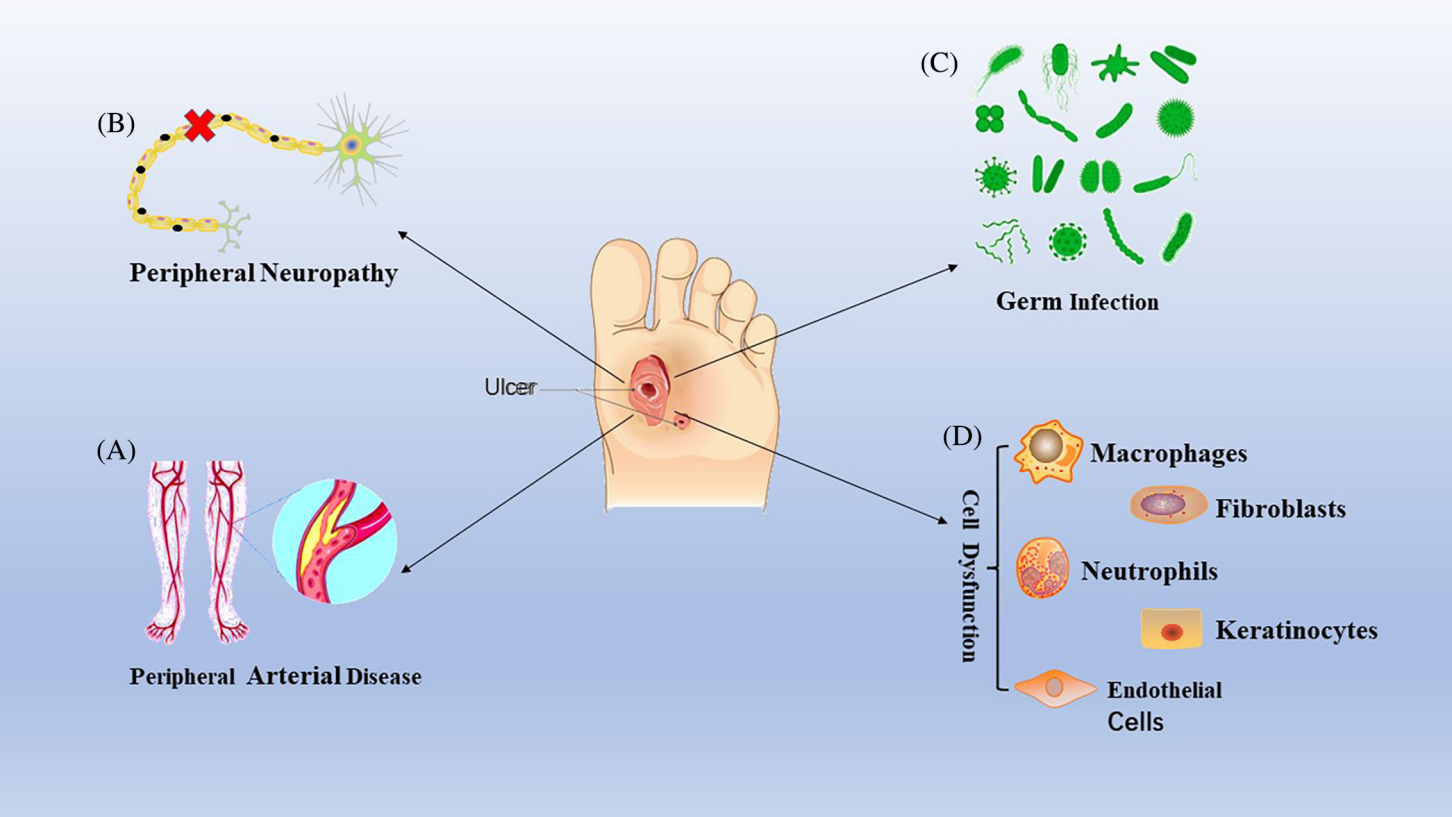
The four main aspects of diabetic foot ulcer formation: peripheral arterial disease, peripheral neuropathy, bacterial infection, and cell dysfunction. (A) Peripheral arterial disease is the most important factor in the development of diabetic foot. Severe ischemia of the skin of the lower extremities leads to ulcerated tissues becoming necrotic due to insufficient blood supply. (B) Peripheral neuropathy leads to sensory, motor, and secretory dysfunctions in the skin of the lower extremities. These pathological changes not only alter the physical mechanics of the feet and lead to a loss of protective sensation (direct factors in the formation of ulcer wounds) but also cause dry skin, which is not conducive to the healing of diabetic wounds. (C) Infection of wounds by bacteria further delays healing. Because of a reduction in beneficial inflammatory factors and an increase in harmful inflammatory factors, normal wound healing is delayed. (D) The functional status of wound cells directly determines healing quality. The special microenvironment of diabetic wounds is not conducive to the execution of normal cell functions. For example, in the inflammatory phase, there is an imbalance between the proinflammatory and anti‐inflammatory effects of macrophages and neutrophils. In the proliferation stage, the proliferation and migration of endothelial cells are impaired. In the remodeling stage, fibroblasts differentiate and secrete collagen abnormally. In addition, dysfunction in keratinocyte proliferation and differentiation is observed.

In diabetic wounds, tissue ischemia, hypoxia, and high glucose microenvironment interfere with the progress of these programmed healing stages, resulting in delayed healing or nonhealing of the wounds and several clinical complications.^8^ The key pathogenic factors in the pathogenesis of diabetic foot are displayed in Figure 1. The mechanism and pathogenesis of DFUs are complex and only a few treatment methods can effectively promote healing; thus, there is an urgent need to develop new methods that can not only reduce expenditure but also effectively cure DFUs. However, the key molecular regulatory mechanisms involved in the healing of DFUs are unclear, and additional studies are warranted for a better understanding of this condition.

## 2 REVIEW

### 2.1 Physiological and pathological changes in DFUs

#### 2.1.1 Peripheral vascular lesions dominated by atherosclerosis

Peripheral artery disease is an essential factor of foot ulcers in nearly 50% of patients with diabetes.^9^ Among them, atherosclerosis is the main cause of peripheral vascular disease. The arterial wall consists of several cells and tissues, including endothelial cells, vascular smooth muscle cells (VSMC), fibroblasts, and components of the extracellular matrix (ECM) such as elastin and collagen.^10^ Abnormal carbohydrate, fat, and protein metabolism in diabetes; the increase in reactive oxygen species (ROS); and the activation of inflammatory mediators can lead to vascular endothelial damage.^11^, ^12^ Arterial endothelial cell injury, vascular smooth muscle dysfunction, inflammatory state, blood hypercoagulability, and abnormal changes in platelets are risk factors that promote atherosclerosis.^13^ On the one hand, hyperglycemia, insulin resistance, excessive free fatty acids, and activated advanced glycation end products (AGEs) inhibit the production of nitric oxide synthase (eNOS) and ROS and alleviate oxidative stress. On the other hand, these factors can lead to an increase in proinflammatory factors such as transcription factors, nuclear factor‐κB (NF‐κB), and activating protein‐1. In this highly activated inflammatory state, white blood cells adhere to the inner wall of the artery, migrate and phagocytize fat, and transform into foam cells, thereby promoting the formation of atherosclerotic lesions.^14^ When macrophages and low‐density lipoproteins (LDLs) penetrate the middle layer of the artery to form foam cells, VSMCs migrate to the arterial stria that is rich in foam cells and lead to the synthesis, secretion, and deposit of ECM, thus promoting atherosclerosis.^15^ Moreover, impaired endogenous platelet inhibition in patients with diabetes can readily activate platelets. The activated endothelium produces an excess of adhesion molecules and platelet agonists. The activated platelets stimulate leukocyte recruitment, which is also activated through interaction with platelets and may promote the progression of atherosclerosis and plaque rupture, resulting in thrombosis and vascular occlusion.^16^

Moreover, the extent to which the lower limbs are affected by atherosclerosis in diabetic patients is different from that in nondiabetic patients. In patients with diabetes, the inferior genicular artery (posterior tibial artery and anterior tibial artery) is primarily involved, whereas the femoral and popliteal artery segments (superficial femoral artery and popliteal artery) are involved to a lesser extent. The main iliac artery is usually not involved. Ischemic ulcers or gangrene may occur with the development of tibial artery occlusion or proximal artery occlusion when the arterial perfusion of the foot is inadequate in maintaining the functional integrity of the skin.^17^ Therefore, peripheral angiopathy is the initial cause of DFU and also the primary factor leading to amputation and death. Atherosclerosis is the main pathological process of peripheral vascular disease. Atherosclerotic plaque rupture can induce peripheral arterial thrombosis, especially in a diabetic environment, directly leading to arterial occlusion and lower limb ischemia, which, in turn, leads to the formation of DFUs.

#### 2.1.2 Peripheral neuropathy

Diabetic foot neuropathy is closely related to age, disease course, and the extent of diabetes control. Among diabetic complications, the clinical syndrome triggered by injury to the peripheral and autonomic nervous systems is very common.^18^ Because of the disordered metabolic environment in diabetes, the pathological changes in the nervous system are closely related to the structural and functional disorders of nerve cells. For example, hyperglycemia, high levels of AGE, excessive acylcarnitine, and oxidized LDLs affect motor, sensory, and other functions by destroying the structure and function of nerve cells. Because of the lack of perception of pain symptoms, the risk of trauma in patients with diabetes is significantly increased; thus, skin damage and ulcers may go unnoticed for weeks or months by both patients and physicians alike.^19^ On the one hand, AGEs cause changes in or loss of protein functions; on the other hand, after binding to AGE‐specific receptors (RAGEs), they modify gene expression and mediate intracellular signal transduction, increasing the production of inflammatory mediators and free radicals,^20^ which seriously interfere with the release and transport of neuronal transmitters. Because of excessive substrate and oversaturated delivery system, acetyl‐CoA molecules are converted into acylcarnitine, which can cause high stress response and mitochondrial dysfunction in Schwann cells and dorsal root ganglion (DRG) neurons and induce axonal degeneration, resulting in irreversible damage to the nervous system of diabetic individuals.^21^ The clinical manifestations of motor neuropathy are mainly muscular atrophy of the leg and foot, and motor paralysis and loss of muscle reflex can also be observed. Achilles tendon reflex dysfunction is an early symptom of motor neuropathy.^22^ Autonomic neuropathy often causes vasomotor dysfunction of the lower extremities, resulting in arteriovenous shunts of the cutaneous vascular network of the lower extremities.^23^ Additionally, autonomic neuropathy can cause dysfunction in sweat gland secretion and increase blood perfusion of the deep skin, resulting in skin overheating.^24^ Abnormalities in the secretion of sweat glands in the skin can cause excessive evaporation of sweat, leading to drying of the skin of the foot, which further impairs its protective function and increases the risk of foot ulcers.^25^ The combination of sensory and motor peripheral neuropathies results in uneven foot pressure load and poor gait. Over time, hyperkeratosis of the compressed skin will form a hematoma and break due to neuropathy and increased plantar pressure load, eventually developing into an ulcer that is difficult to heal.^26^

Studies in rodent models reveal that hyperglycemia alters the function of key plastic molecules of the nervous system, including the expression patterns of neuromodulin, β‐tubulin, heat‐shock protein, and poly‐ADP‐ribose polymerase in DRG.^27^, ^28^ Abnormalities in DRG function, including the change in splice body function, change of motor neuron protein expression, and the upregulation of GW‐body (mRNA processing site), are important links in diabetic neuropathy.^29^

In addition, plasma LDLs in patients with diabetes are oxidized by ROS, after which they bind to oxidized LDL receptors 1 and 4 and RAGE. This, in turn, activates a series of signaling pathways, including caspase 3 and ribonucleic acid pathways, mediating additional inflammatory responses and leading to the accumulation of reactive oxygen free radicals, which can cause irreversible nerve tissue damage.^30^, ^31^ Some studies have found disorders in polyol and inositol metabolism: Na/K‐adenosine triphosphate (ATP) enzyme degradation, neurovascular defects, neurotrophic disorders, axonal transport defects, and nonenzymatic glycosylation of neurons and transport proteins in the nerves of diabetic individuals.^32^, ^33^ Pathological changes in these key pathways can lead to abnormal protein processing, oxidative damage, and mitochondrial dysfunction in neurons, leading to the loss of peripheral nerve function.^34^

#### 2.1.3 Wound infections

Diabetic foot infection is a common and serious complication in patients with diabetes. Insufficient arterial blood supply to the lower extremities and multiple neuropathies increase the risk of diabetic foot infection. DFIs are usually caused by a single bacterium (*Staphylococcus aureus*). Chronic infections may be to the result of mixed infections by several microorganisms, including not only *S. aureus* and *Streptococcus* spp., but also coagulase‐negative staphylococci, enterococci, gram‐negative bacteria, and anaerobes.^35^ In addition, the most common fungal pathogens responsible for DFIs are *Candida albicans*, *candida subsmooth*, and *C. tropicalis*. Among them, *candida subsmooth* is the most common yeast strain isolated from DFU tissue because it is often isolated from under the nails.^36^ This mixed infection poses special challenges in the treatment of patients with DFUs especially when complicated by bacterial and fungal infections. Persistent DFIss are closely related to immune cell dysfunction, including impaired immune function (leukopenia) and inflammatory response disorder (persistent inflammation of macrophages),^37^ which are discussed subsequently.

##### Changes in the primary cellular biological functions in patients with DFUs

**FIGURE 2:**
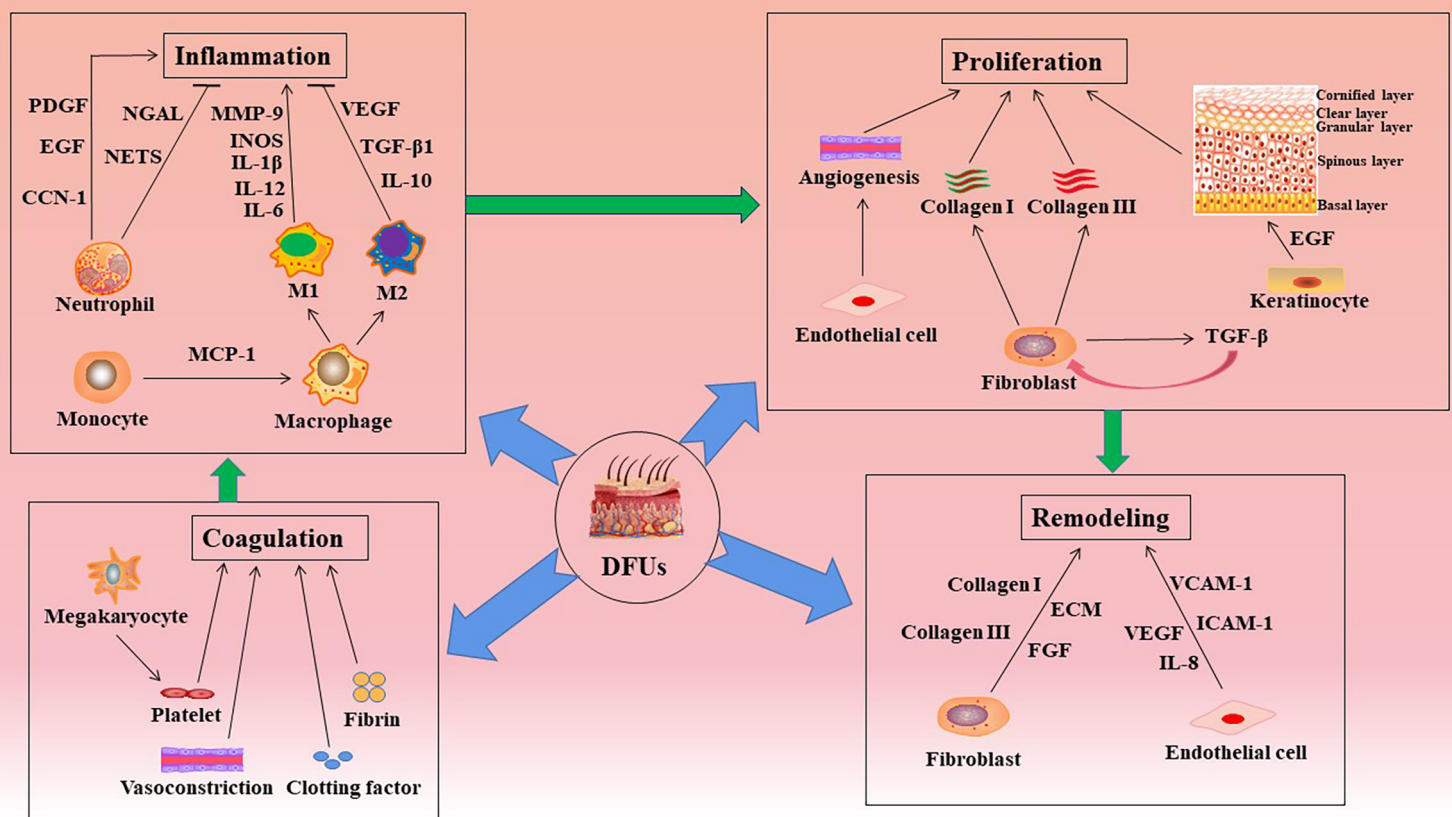
The process of wound healing generally needs to go through the stages of coagulation, inflammation, proliferation and remodeling. Different cells are involved in each stage. In addition, cells at different stages secrete a variety of cytokines to speed up the healing process. CCN‐1, cellular communication network factor‐1; DFU, diabetic foot ulcer; ECM, extracellular matrix; EGF, epidermal growth factor; FGF, fibroblast cytokine; ICAM, intracellular adhesion molecule; IL, interleukin; iNOS, inducible nitric oxide synthase; MCP‐1, monocyte chemoattractant protein‐1; MMP‐9, matrix metalloproteinase‐9; NET, neutrophil extracellular trap; NGAL, neutrophil gelatinase‐associated lipocalin; PDGF, platelet‐derived growth factor; TGF‐ β1, transforming growth factor beta‐1; VCAM, vascular cell adhesion molecule; VEGF, vascular endothelial growth factor.

Immune cells, keratinocytes, fibroblasts, endothelial cells, and different cytokines are involved in the healing of DFUs. In the inflammatory stage, infiltrating monocytes/macrophages in the wound are essential for the transformation of the wound from a proinflammatory to an anti‐inflammatory environment.^38^ In the later stage of normal wound inflammation, macrophages change from the proinflammatory to the anti‐inflammatory phenotype.^39^ However, in individuals with DFUs, the macrophage function/phenotypic transition is impaired and macrophages continue to maintain a proinflammatory state.^40^ Clinical and experimental evidence shows that, contrary to that observed in normal tissue, healing of DFUs is characterized by leukocyte recruitment, macrophage activation, and the production of proinflammatory cytokines, showing a chronic proinflammatory state.^41^ In addition, the phagocytic ability of macrophages in DFU wounds is significantly impaired and ineffective in removing necrotic tissue from the wound.^42^ In addition to macrophage response, the inflammatory response is enhanced by neutrophils and can adversely affect the healing of diabetic wounds. In diabetic wounds, phagocytosis, neutrophil degranulation, and the anti‐infective effects of ROS are disrupted.^43^ Excessive infiltration and activation at the site of tissue injury mediate tissue damage by releasing cytokines and proteases and regulating the adaptive immune response.^44^ Moreover, the expression of neutrophil protein arginine deiminase (PAD)‐4 is upregulated by hyperglycemia. When neutrophils invade the wound, their ability to secrete NETs is inhibited, resulting in delayed wound healing.^45^, ^46^ Macrophages and neutrophils secrete proteases in the form of zymogen, which is activated outside the cell and degrades ECM proteins (elastin and interstitial collagen). For example, MMP degrades fibronectin into fragments and further activates MMPs. These fibronectin fragments cause leukocyte infiltration, tissue damage, and persistent inflammation.^47^ Therefore, different cells will play a therapeutic role with unique functions in diabetic wound healing (Figure 2).

In the healing stage of DFUs, reepithelialization and dermal repair play a vital role in skin tissue regeneration.^48^ Studies have shown that during wound reepithelialization, keratinocytes migrate to the wound site and proliferate and differentiate into different structures to restore the integrity of the structure and function of the epidermis.^49^ The order followed from the inside to the outside is skin epidermis, basal cell layer, spinous cell layer, granular cell layer, and stratum corneum. In the early stage of wound healing, keratinocytes can directly kill invasive pathogens by secreting cytokines, chemokines, antimicrobial peptides, and extracellular vesicles to mediate the interaction between keratinocytes and circulating immune cells, thereby promoting wound healing.^50^ However, the high glucose environment of diabetic wounds disrupts the normal functioning of keratinocytes, resulting in delayed wound reepithelialization. Dermal repair is mainly achieved by the proliferation, differentiation, and secretion of ECM components of fibroblasts.^51^ Special fibroblasts in the upper (papillary) layer can form hair papillae and regulate hair follicle growth and hair regeneration. Lower layer (reticular) fibroblasts are primarily involved in maintaining the morphological structure of the dermis, providing a stable environment for angiogenesis, nerve regeneration, and immune‐clearance activities.^52^ Additionally, fibroblasts can differentiate into myofibroblasts that promote wound contraction, secrete proteases and MMPs to degrade the inflammatory matrix, and secrete collagen and other ECM proteins to participate in the formation of granulation tissue. Collagen III in the ECM is replaced by collagen I, which has a higher tensile strength.^53^ However, hyperglycemia and the accumulation of AGEs lead to impaired fibroblast function, such as decreased proliferation, accelerated apoptosis, and inhibition of migration to the wound site, all of which lead to impaired dermal repair and delayed healing of diabetic wounds.^54^, ^55^

Endothelial cell quality and neovascularization play a vital role in wound healing. Endothelial cells are generally arranged on the inner surface of the vascular lumen and regulate vasoconstriction and dilatation by modulating the levels of vasoactive factors such as eNOS.^56^ In wound healing, endothelial cells at different stages of angiogenesis are mainly regulated by VEGF. In the inflammatory stage, VEGF increases vascular permeability, affects the expression of selectin and intercellular adhesion molecules in endothelial cells, and promotes the recruitment of leukocytes to the injured site. In the proliferation stage, VEGF strongly stimulates the proliferation and migration of endothelial cells, whereas, in the remodeling stage, it induces endothelial cell assembly to promote vascular lumen formation.^57^ In vivo studies have shown that arterial endothelial cells in a hyperglycemic environment lose their integrity. They are more prone to apoptosis and to shedding and entering the blood circulation, leading to angiogenesis disorders.^58^ This damage is mainly attributed to the following five pathways: (a) polyol pathway, (b) increase of intracellular AGEs, (c) upregulation of RAGE, (d) activation of various subtypes of protein kinase C, and (e) overactivation of the hexosamine pathway.^59^ In diabetes, the decrease in NOS due to peripheral neuropathy and peripheral arterial disease leads to a decrease in peripheral blood flow by vasoconstriction. The lack of endothelial progenitor cells (EPCs) in the wound inhibits the formation of new blood vessels and delays wound healing.^60^

Stem cells are the critical cells in postinjury and routine homeostasis skin repair in the healing of DFU. Stem cells have the characteristics of asymmetric replication, the potential of strong self‐renewal, and multidifferentiation.^61^ In particular, the functional state of EPCs and epidermal stem cells (ESCs) critically influences the process of wound healing. EPC functions such as migration, differentiation, adhesion, and tube formation are impaired in the hyperglycemic state of diabetes,^62^ which causes long‐term wound nonhealing, particularly in chronic wounds like diabetic wounds.^63^ As the precursor of endothelial cells, EPCs migrate from bone marrow to peripheral blood under the action of hypoxia inducible factor‐1, stromal cell derived factor‐1α, and VEGF and are recruited to the ischemic site to form new blood vessels through adhesion, proliferation, differentiation, and tube formation to repair wounds.^64^ Moreover, ESCs also play an indispensable role in process of wound healing. In vitro experiments show that ESCs enhance the proliferation and migration of diabetic fibroblasts and macrophages (Mφ), and promote alternative or M2 Mφ polarization. In wounds of db/db mice, treatment with ESCs accelerate wound healing by decreasing inflammation, augmenting wound cell proliferation, stimulating angiogenesis, and inducing M2 Mφ polarization.^65^

In recent years, intercellular communications play an important role in the development of many diseases. As an important mediator of cellular communication, exosomes carry and transmit important signaling molecules and are widely involved in intercellular material transport and information transfer; they regulate cellular physiological activities and are closely related to the occurrence and course of various diseases.^66^ Under physiological and pathological conditions, almost all cells can produce and secrete exosomes, including immune cells (such as B cells, T cells, mast cells, dendritic cells), platelets, cancer cells, epithelial cells, mesenchyme cells, neurons, astrocytes, and oligodendrocytes.^67^, ^68^ Exosomes are widespread and distributed in various body fluids, they are rich in nucleic acids (microRNA, lncRNA, circRNA, mRNA, tRNA, etc.), proteins, lipids, etc.^69^ Mesenchymal stem cells (MSC)‐exosomal ncRNAs have shown great potential in skin healing as it promotes wound healing. A study by Liang et al found that human adipose‐derived MSC secreted exosomes (adMSC‐Exo) could translocate microRNA‐125a (miR‐125a) to endothelial cells and promote angiogenesis by inhibiting delta‐like 4 (DLL4). This may have a function in wound repair in DFUs.^70^ Additionally, Xu Juan et al found that miRNA‐221‐3p was highly expressed in EPC‐derived exosomes and promoted wound healing in diabetic mice.^71^ Moreover, a study by Qijun Lu et al found that engineered human adipose stem cell‐derived exosomes containing miR‐21‐5p could promote keratin formation and cell proliferation and migration via Wnt/β‐catenin signaling in vitro and increase epithelial reformation, collagen remodeling, angiogenesis, and maturation in vivo to promote diabetic skin wound healing.^72^ However, there are still many challenges in applying exosomal ncRNAs in actual clinical treatment. With further research, exosomal ncRNAs will show great value in the prevention, diagnosis, and treatment of diabetic foot.

##### The role of the main signaling pathway of DFU

**FIGURE 3:**
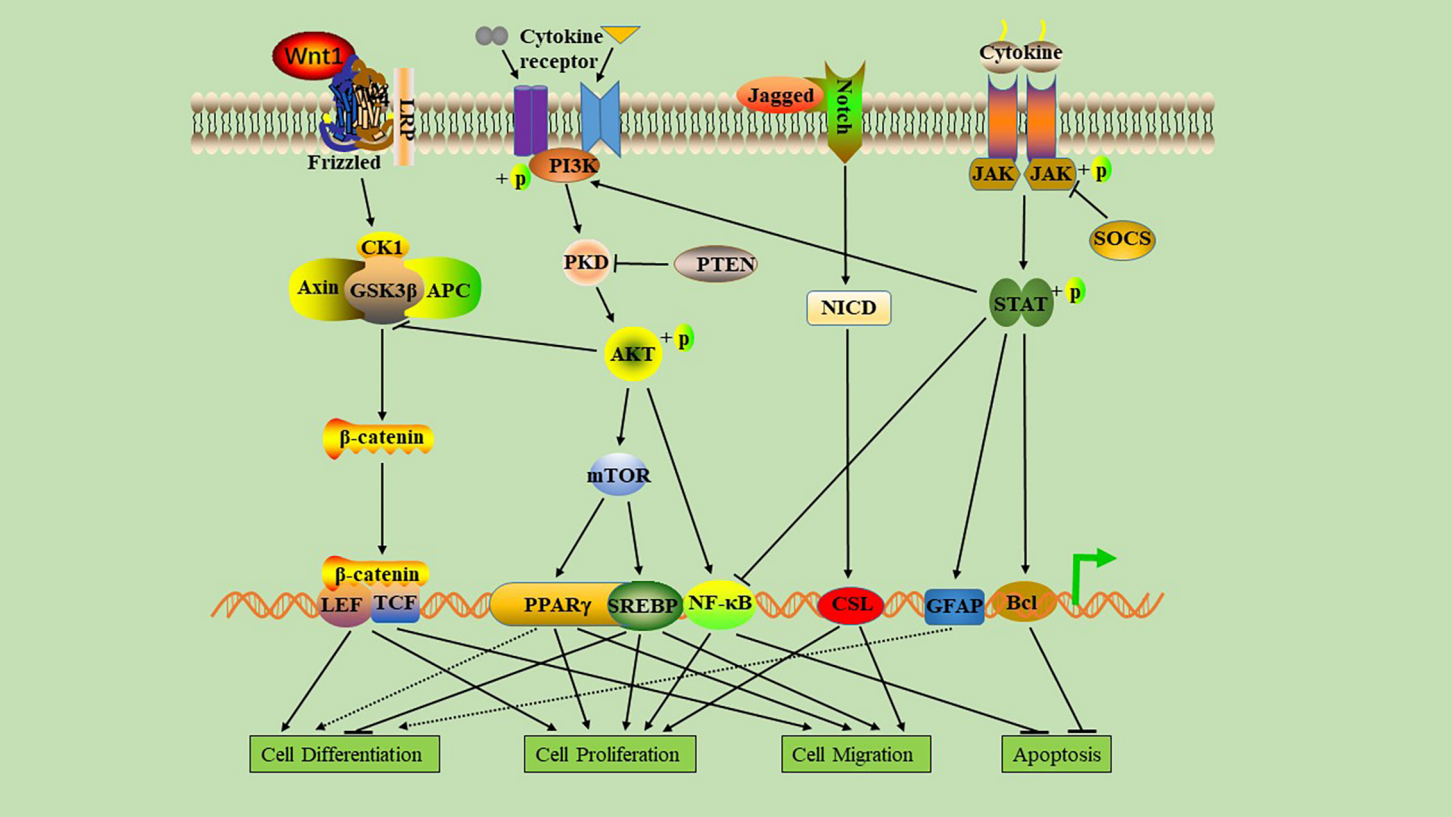
Regulatory role of several key signaling pathways in wound healing. Activation of the Wnt/β‐catenin signaling pathway can promote cell proliferation, migration, and differentiation. Activation of the phosphatidylinositol‐3‐kinas (PI3K)/Akt/mammalian target of rapamycin (mTOR) signaling pathway stimulates cell proliferation, migration, and differentiation, and inhibits autophagy. It also affects the function of Wnt/β‐catenin through phosphorylated Akt. This pathway can be inhibited by PTEN. Activation of the Notch signaling pathway can promote cell proliferation and migration. Activation of the Janus kinase and signal transducer and activator of transcription (JAK–STAT) signaling pathway can promote cell differentiation and inhibit autophagy, and phosphorylated STAT can enhance the activation of PI3K and inhibit the function of NF‐κB. However, this signaling pathway can be inhibited by suppressor of cytokine signaling (SOCS). The NF‐κB signaling pathway can promote cell proliferation and inhibit autophagy. AKT, protein kinase B; APC, adenomatous polyposis coli; CK1, casein kinase 1; CSL, C‐promoter binding factor‐1; GFAP, glial fibrillary acidic protein; GSK3β, glycogen synthase kinase‐3 beta; LEF, lymphoid enhancer binding factor; NF‐κB, nuclear factor‐κB; NICD, Notch intracellular domain; PKD, protein kinase D; PPARγ, proliferator‐activated receptor gamma; SREBP, sterol regulatory element‐binding protein; TCF, T cell factor.

In the human life cycle, signaling pathways are involved in the occurrence, development, and outcome of various diseases. Signaling pathways have both positive and negative regulatory effects. Whether positive regulation or negative regulation, these pathways are involved not only in disease progression but also in disease treatment. Moreover, several signaling pathways cross‐regulate each other, and this complex network of regulation brings infinite possibilities to research. In the next section, we explore, in detail, the regulatory role of important signaling pathways involved in diabetic wound healing (Figure 3).

#### 2.1.4 Role of the phosphatidylinositol‐3‐kinas/Akt/mammalian target of rapamycin (PI3K/Akt/mTOR) signaling pathway in the healing process of DFUs

The PI3K/Akt/mTOR signaling pathway is involved in regulating the occurrence and recovery of many diseases and plays a role in the migration, proliferation, differentiation, and apoptosis of repaired cells in the healing of diabetic ulcers. Studies have found that the PI3K/Akt/mTOR pathway can regulate the accumulation of lipids in endothelial cells and fibroblasts to prevent diabetic wound cell fading and enhance wound healing.^73^, ^74^ When the PI3K/Akt/mTOR signal pathway is activated, it can upregulate the expression of VEGF, FGF, and EGF; promote proliferation, migration, angiogenesis, and collagen synthesis; and induce epithelial‐mesenchymal transition (EMT) and stimulate wound healing.^75^, ^76^ Activation of the PI3K/Akt/mTOR signaling pathway not only enhances the mRNA expression of alpha smooth muscle actin, fibronectin, collagen (COL1A1, COL3A1), and transforming growth factor beta but also promotes the proliferation and differentiation of fibroblasts, thereby accelerating wound closure.^77^ In the wounds of individuals with hyperglycemia, protein kinase B (AKT) phosphorylation can directly phosphorylate eNOS and induce NO production after activation of the PI3K/AKT‐eNOS pathway, serving as a key regulator of vascular protection, maintaining endothelial cell function, and promoting angiogenesis, thus indicating its role in diabetic wound healing.^78^

#### 2.1.5 Role of Wnt/ β‐catenin signaling pathway in the healing of DFUs

The Wnt/β‐catenin pathway is a very conservative signaling pathway that participates in several biological processes such as inflammation, carcinogenesis, fibrosis and angiogenesis, cell proliferation, apoptosis, and differentiation. It is also an important signaling pathway that regulates wound healing.^79^, ^80^ Healing is accelerated in normal wounds when the Wnt/β‐catenin pathway is upregulated and the proliferation, differentiation, and migration of epidermal cells are enhanced.^81^ In DFUs, downregulated Wnt/β‐catenin signaling leads to suppression of the biological activity of skin cells and the expression of cytokines, resulting in immune dysfunction of the wound, dysplasia of granulation tissue, and reepithelialization disorders, thus delaying wound healing.^82^ In addition, pigment epithelium‐derived factor in diabetic wounds inhibits Wnt/β‐catenin signaling, resulting in mobilization and dysfunction of EPCs, thereby inhibiting angiogenesis and delaying wound healing.^83^ In the wound‐healing stage of ulcers, macrophages are closely related to the Wnt/β‐catenin pathway. Wnt5a affects the inflammatory response by regulating macrophage phenotype. Wnt7b is also a key protein that promotes wound angiogenesis.^84^ Some studies show that downregulation of the Wnt/β‐catenin pathway may be related to a decrease in Rspo protein caused by diabetes.^85^ Studies have shown that hyperglycemia affects the proliferation, migration, and collagen secretion of fibroblasts through phosphorylation of β‐catenin and its accumulation in the cytoplasm, migration to the nucleus, and regulation of target gene transcription.^86^

#### 2.1.6 Role of Notch signaling pathway in the healing of DFUs

The Notch pathway plays a key role in cell differentiation, proliferation, and angiogenesis and participates in the regulation of tissue homeostasis in humans. In diabetic wounds, Notch pathway dysfunction can lead to inhibition of cell differentiation, proliferation disorders, and reduced angiogenesis, all of which interfere with diabetic wound healing.^87^ Some studies report that Notch1 activation leads to impaired proliferation of keratinocytes, whereas a defect in Notch1 signaling in keratinocytes leads to excessive epidermal proliferation in mice that may even result in skin tumors.^88^ Hyperglycemia directly promotes the pathological activation of Notch1 signals in the skin of diabetic individuals by activating the positive feedback loop between the Dll4 ligand and Notch1 receptor, which inhibits diabetic wound healing.^89^ Moreover, Notch signaling is closely related to the development and differentiation of monocytes and macrophages.^90^ In DFUs, high levels of kallikrein binding protein activate the Notch signal pathway, promote macrophage polarization to M1, increase the number of macrophages in the wound, and lead to a pronounced inflammatory response, which slows down wound healing in patients with diabetes.^91^

#### 2.1.7 Role of Janus kinase and signal transducer and activator of transcription (JAK–STAT) signaling pathway in the healing of DFUs

The JAK–STAT signaling pathway is essential in maintaining balance in the body.^92^ In diabetic individuals, growth factors and chemokines can activate the JAK–STAT pathway. When the STAT protein is phosphorylated by JAK protein, as it constitutes an essential step to ensure dimerization, nuclear translocation, DNA attachment, and transcription of target genes.^93^ A recent study found that the expression of suppressor of cytokine signaling 3 (SOCS3) in diabetic wounds decreased significantly. Moreover, during most of the wound‐healing period, the expression of interleukin‐6 (IL‐6) and IL‐6R α increased significantly, resulting in a sharp increase in phosphorylated STAT3 levels, leading to the dysfunctional inflammation of diabetic wounds.^94^ In addition, the gp130/JAK–STAT3 signal loop mediated by IL‐6R is negatively regulated by SOCS3. SOCS3 is expressed in the epithelium of the wound and its overexpression delays the healing of diabetic wounds by interfering with the proliferation and migration of keratinocytes.^95^ In DFUs, the upregulation of forkhead box protein M1 (FOXM1) and STAT3 mediates impaired immune cell activation, recruitment, and survival, resulting in delayed wound healing.^96^ Photobiological regulation at 660 nm promotes the secretion of EGF and EGF receptor (EGFR) in diabetic wounds and activates the JAK/STAT signaling pathway in fibroblasts, leading to the migration and proliferation of downstream cells, which promote wound healing.^97^ In short, JAK–STAT mediates the biological activity of various cytokines/chemokines; thus, the regulation of JAK–STAT signaling is a promising target for the treatment of diabetic wounds.

#### 2.1.8 Role of NF‐κB signaling pathway in the healing of DFUs

The NF‐κB pathway regulates hundreds of genes that are involved in several important cellular responses, such as inflammation, cell migration, proliferation, and apoptosis.^98^ Moreover, NF‐κB regulates the expression of proinflammatory genes in macrophages and granulation area and enhances the expression of degrading enzymes in cytokine synthesis, including cytokines, chemokines, and adhesion molecules.^99^ The activated NF‐κB complex is transferred to the nucleus, where it binds to the DNA in the B‐binding motif to promote the expression of proinflammatory enzymes and proinflammatory factors such as IL‐6, tumor necrosis factor (TNF)‐α, and inducible NOS.^100^ In addition, inflammatory disorders mediated by NF‐κB can lead to wound inflammation and neurodegenerative diseases.^101^ High glucose levels can induce the activation of NF‐κB, promote apoptosis of human endothelial cells by enhancing caspase‐3 activity, and delay wound healing in individuals with diabetes mellitus.^102^ Some studies have confirmed that negative pressure wound therapy can inhibit the release of proinflammatory enzymes and cytokines by preventing the activation of NF‐κB, thereby increasing wound‐healing rate, reducing healing time, and lowering the risk of amputation.^103^ Hyperbaric oxygen therapy activating the NF‐κB signal can promote stromal cell derived factor‐1 and VEGF expression in fibroblasts, regulate cell proliferation and migration, and promote angiogenesis and wound healing.^57^ The NF‐κB signaling pathway is one of the key pathways of nuclear signal regulation and its functional changes in DFUs directly determine wound healing. However, there is limited clarity regarding the key molecules that determine the activation or inhibition of NF‐κB signaling pathway; thus, in‐depth studies are required.

##### Epigenetic changes in DFUs

DFU is a severe complication of diabetes and epigenetics has been shown to play a key regulatory role. There are three main types of epigenetic gene regulation: DNA modification, biochemical modification of histone tail, and ATP‐dependent chromatin remodeling.^104^ For example, DNA methylation caused by long‐term hyperglycemia and noncoding RNA modification caused by gene–environment interaction can lead to complications.^105^ Therefore, in‐depth studies on the epigenetic mechanism of DFUs will greatly improve the current situation and shed light on the healing process. The subsequent sections focus on the epigenetic regulatory mechanisms of the primary cells involved in the healing of DFUs, including the effects of gene methylation, demethylation, and histone modification in inflammatory cells, keratinocytes, fibroblasts, and vascular endothelial cells.

#### 2.1.9 Epigenetic regulation of inflammatory cells

Some studies have shown the effects of DNA methylation and demethylation on the function of inflammatory cells in diabetic wounds. For example, H3K27 demethylase and Jumonji‐domain‐containing protein D3 (JMJD3) have been shown to play a role in the activation of pro‐ and anti‐inflammatory macrophage phenotypes. In mouse macrophages, JMJD3 can be upregulated by lipopolysaccharide and IL‐4 to promote the expression of proinflammatory genes or IL‐4 target genes.^106^ It has been reported that the inhibition of DNA methyltransferase‐1 (DNMT1) by 5‐azacytidine promotes the formation of M2 macrophages and inhibits the inflammation of bone marrow‐derived macrophages (BMDMs).^107^ DNMT1 in BMDMs increase and enhance the production of proinflammatory macrophage phenotype in a type 2 diabetes mouse model of genetically modified and diet‐induced obesity.^108^ Wound healing in knockout DNMT1 and db/db mice has shown significant improvement. In the inflammatory stage of wound healing, mixed leukemia gene (MLL)1 has been shown to promote the deposition of H3K4me3 in macrophages and reduce the inflammatory response in diabetic wounds. Thus, MLL1 plays an important role in regulating macrophage‐mediated inflammation in wound healing.^109^ In the diet‐induced obesity model of diabetes, macrophages promote the methylation expression of JMJD and H3K4, which in turn reduces the methylation of H3K27. Among them, the increase in JMJD3 levels has been indicated as the likely reason for the decrease in H3K27 methylation.^110^ JMJD3 stimulates IL‐12 expression in wound macrophages, which can delay wound healing.^106^ Neutrophil activity in the healing of DFUs is also regulated by epigenetics. During NETosis, neutrophils are activated by PAD‐4 and undergo histone citrullination, resulting in exocytosis and apoptosis of the complex formed by NETs. Although this process protects against infection by engulfing the bacteria, the release of NETosis can interfere with wound tissue healing. In fact, the typical granule components of NETs such as neutrophil protease3, neutrophil elastase, and myeloperoxidase; and nuclear components such as histones H2A, H2B, and H3 increase in nonhealing diabetic wounds. Thus, the epigenetic regulation of neutrophils is closely related to the interference with wound healing.^111^

#### 2.1.10 Epigenetic regulation of keratinocytes and fibroblasts

Abnormal DNA methylation has been confirmed in keratinocytes and fibroblasts in DFUs in humans and is closely related to reepithelialization, angiogenesis, and extracellular matrix precipitation.^112^ For example, JMJD3 promotes the migration of keratinocytes to the wound site by increasing Notch1 expression.^113^ Tumor necrosis factor‐α promotes MMP9 expression in keratinocytes, depending on the specific demethylation of MMP9 promoter, thereby delaying the migration and proliferation of keratinocytes, which is closely related to the nonhealing of diabetic wounds.^114^ In addition, ASH1L‐targeting histone H3 is related to the methylation of K4, K9, K20, and K36 at the active gene promoter.^115^ The absence of Ash1l hinders the reepithelialization of normal wounds.^116^ Expression of the miR‐197‐3p target caveolin‐1 in fibroblasts is significantly upregulated in unhealed DFU tissues. Inhibiting caveolin‐1 expression and activating the EGFR pathway can stimulate the migration and proliferation of fibroblasts and accelerate wound healing.^117^ Therefore, reprogramming the diabetic fibroblasts inhibits the expression of negative regulatory factors through the regulation of miR; thus, mature fibroblasts can promote the epigenetic characteristics of wound healing.^118^

#### 2.1.11 Epigenetic regulation of vascular endothelial cells

In DFU wounds, an increase in cellular ATP can stimulate wound healing by increasing angiogenesis and collagen production.^119^ Human mammary epithelial cells cultured in high glucose conditions show epigenetic changes that increase the expression of the NF‐κB‐subunit p65 gene, induce the secretion of vascular cell adhesion molecule‐1, mediate leukocyte‐endothelial cell adhesion, and finally promote the development of atherosclerotic lesions of the vascular wall.^120^ This epigenetic change is due to H3K4 methylation and H3K9 demethylation of lysine‐specific demethylase‐1 in the NF‐κB‐p65 promoter, which leads to the continuous open state of NF‐κB‐p65 transcriptional activity.^121^ In addition, the increased transcription of NF‐κβ‐p65, monocyte chemoattractant protein‐1, and IL‐6 in the aortic endothelial cells of patients with DFUs can lead to the overexpression of miR‐125b and downregulation of miR‐146a‐5p, which is a regulator of endothelial dysfunction in metabolic memory. Thus, miR‐125b and miR‐146a‐5p may be explored as novel targets to promote wound healing in diabetic individuals.^122^ During wound healing, endothelial cells secrete a large amount of miR‐191. As a downstream effect, the miRNA targets the silencing small band‐1 gene, which encodes a multidomain functional protein that regulates cell adhesion, tissue structure maintenance, angiogenesis, and cell migration during tissue repair, resulting in difficulties in wound healing in individuals with diabetes.^123^

### 2.2 Future perspectives

Currently, there is great potential and significant room for improvement with respect to the pathogenesis and treatment of DFUs. First, it is necessary to clearly understand the pathogenesis of DFUs as it is the cornerstone of curing this condition. Only by accurately comprehending the regulation of the pathogenesis of DFUs can new drugs or interventions for relevant targets be developed.

Additionally, the use of stem cells to treat DFUs is emerging as a promising treatment. Embryonic and mesenchymal stem cells can be induced to differentiate into myofibroblasts, keratinocytes, and endothelial cells, which are components of wound healing.^124^ At the same time, stem cells synthesize and secrete several cytokines to promote cell recruitment, immune regulation, ECM remodeling, angiogenesis, and nerve regeneration.^125^ However, unlimited cloning and proliferation of stem cells pose great challenges, such as the formation of skin tumors, in clinical treatment. We always firmly believed that based on the unremitting efforts of researchers worldwide, stem cell proliferation and differentiation will eventually be accurately regulated and streamlined.

However, reverse differentiation of cells also shows promise in the clinical treatment of DFUs. Our research team found that keratinocytes can reverse‐differentiate into embryonic progenitor cells when induced by basic fibroblasts and can participate in angiogenesis, reepithelialization, and ECM precipitation.^126^, ^127^ These findings have been partially verified in cell‐based experiments as well as in mice.

## 3 CONCLUSIONS

To summarize, we need to devote ourselves to research on the pathogenesis of DFUs at different levels, constantly explore new key regulatory targets, develop novel products for use in a clinical setting, and identify measures that could promote the healing of DFUs.

## AUTHOR CONTRIBUTIONS

Conception and design: Haibo Deng, Binghui Li; collection and assembly of data: Haibo Deng, Chenchen Zhang, Liewn Kuang, SiYuan Wang, ZhiQiang Ma; data analysis and interpretation: Haibo Deng, Qian Shen, Ran Chen, Gongchi Li; graphic illustration: Haibo Deng; manuscript writing: all authors; manuscript revision: Haibo Deng, Gongchi Li; final approval of manuscript: all authors.

## FUNDING INFORMATION

This work was supported by Building Project of Hubei Natural Science Foundation Project (2020CFB696) and Medical letter of the State Health Office (National Natural Science Foundation of China [81801922] and Research and Development Program of Hubei Province [2020BCB029]).

## CONFLICT OF INTEREST STATEMENT

None declared.

## ETHICAL APPROVAL

No human or animal studies involved or no ethical statement for the study.
